# Aquarius is required for proper CtIP expression and homologous recombination repair

**DOI:** 10.1038/s41598-017-13695-4

**Published:** 2017-10-23

**Authors:** Ryo Sakasai, Mayu Isono, Mitsuo Wakasugi, Mitsumasa Hashimoto, Yumi Sunatani, Tadashi Matsui, Atsushi Shibata, Tsukasa Matsunaga, Kuniyoshi Iwabuchi

**Affiliations:** 1 Department of Biochemistry I, Kanazawa Medical University, Ishikawa, Japan; 2 Education and Research Support Center, Gunma University, Ishikawa, Japan; 3 Faculty of Pharmacy, Institute of Medical, Pharmaceutical and Health Sciences, Kanazawa University, Ishikawa, Japan; 4 Department of Physics, Kanazawa Medical University, Ishikawa, Japan

## Abstract

Accumulating evidence indicates that transcription is closely related to DNA damage formation and that the loss of RNA biogenesis factors causes genome instability. However, whether such factors are involved in DNA damage responses remains unclear. We focus here on the RNA helicase Aquarius (*AQR*), a known R-loop processing factor, and show that its depletion in human cells results in the accumulation of DNA damage during S phase, mediated by R-loop formation. We investigated the involvement of Aquarius in DNA damage responses and found that *AQR* knockdown decreased DNA damage-induced foci formation of Rad51 and replication protein A, suggesting that Aquarius contributes to homologous recombination (HR)-mediated repair of DNA double-strand breaks (DSBs). Interestingly, the protein level of CtIP, a DSB processing factor, was decreased in *AQR*-knockdown cells. Exogenous expression of Aquarius partially restored CtIP protein level; however, CtIP overproduction did not rescue defective HR in *AQR*-knockdown cells. In accordance with these data, Aquarius depletion sensitized cells to genotoxic agents. We propose that Aquarius contributes to the maintenance of genomic stability via regulation of HR by CtIP-dependent and -independent pathways.

## Introduction

Cells harbour several systems to counter various types of DNA damage, including DNA double-strand breaks (DSBs) and DNA replication stress, which trigger DNA damage responses (DDRs) such as DNA repair, cell cycle arrest, and apoptosis. In response to DSBs, DNA damage sensors such as ataxia-telangiectasia mutated (ATM) and DNA-dependent protein kinase (DNA-PK) are activated and convey damage signals to downstream factors that promote DSB repair through non-homologous end-joining (NHEJ) and homologous recombination (HR)^1–3^. Although the majority of DSBs induced by ionizing radiation or radiomimetic drugs can be repaired by NHEJ, some are repaired by HR during S and G2 phases. HR is an error-free system to repair DSBs, in which the sister chromatid is used as an intact template for copying the homologous sequence to seal the broken strands. HR is available for repair not only of DSBs but also of stalled replication forks. Mechanistic details of the initial reactions in HR have emerged in recent years. In brief, the DSB processing factor CtIP initiates DNA end resection to expose single-strand DNA (ssDNA) at DSB ends, in cooperation with other proteins including Mre11 and Exo1 nucleases^4,5^. After resection, replication protein A (RPA) heterotrimeric complexes are loaded onto ssDNA, which is followed by loading of Rad51, an HR factor responsible for strand pairing and exchange of homologous sequences^6^. Thus, RPA nuclear foci and their phosphorylation have frequently been used as a marker of DNA end resection, and Rad51 foci have been used as a marker of ongoing HR. ssDNA exposure also elicits activation of the other damage sensor, ATM- and Rad3-related (ATR), leading to downstream Chk1 phosphorylation^7^.

In addition to the well-established pathway of DDR described above, several reports indicate that transcription or RNA is also involved in damage responses. HR frequency is elevated at transcriptionally active sites, a phenomenon termed transcription-associated recombination^8–10^. Thus, it has been proposed that a collision between a replication fork and the transcription machinery causes replication or transcription stalling, which elevates HR frequency. In other instances, dysfunction of RNA export factors or pre-mRNA splicing factors causes transcription-associated DNA damage, leading to hyper-recombination in yeast and human cells^11–15^. The putative structure commonly underlying these phenomena is the R-loop, which is formed by hybridization of nascent RNA to template DNA. The failure of RNA biogenesis, including RNA export and splicing, leads to transcription stalling and the consequent accumulation of R-loops, which subsequently causes DNA damage and genomic instability via interference with DNA replication^13,16,17^.

On the other hand, several R-loop processing factors have been identified. Senataxin is a well-studied DNA-RNA helicase that resolves R-loops. Loss of senataxin causes R-loop accumulation in yeast and human, and elevates recombination frequency in yeast^18,19^. Senataxin also reportedly associates with replication forks and resolves the conflict between replication and transcription by processing R-loops^20^. In addition to senataxin, Sollier *et al*. recently reported Aquarius as a new factor for R-loop processing^21^. Also known as IBP160, Aquarius is a pre-mRNA splicing factor and has RNA helicase activity^22,23^. Aquarius appears to form complexes with other splicing factors such as PRP19 and XAB2 that are also known to be involved in DDRs^22,24,25^. In the absence of Aquarius, accumulated R-loops are resolved in pathways that are dependent on the transcription-coupled nucleotide excision repair (TC-NER) factor CSB (Cockayne syndrome group B) and the NER endonuclease XPF (xeroderma pigmentosum group F)^21^.

In this study, we focus on Aquarius and show that R-loop-mediated DNA damage accumulates in S phase and that the HR pathway is disrupted in Aquarius-depleted cells. In addition, Aquarius depletion causes CtIP downregulation and sensitizes cells to DNA-damaging agents. These results suggest that Aquarius is required for the maintenance of genome integrity via CtIP-dependent and -independent pathways.

## Results

### Aquarius prevents R-loop-derived DNA damage

**Figure 1:**
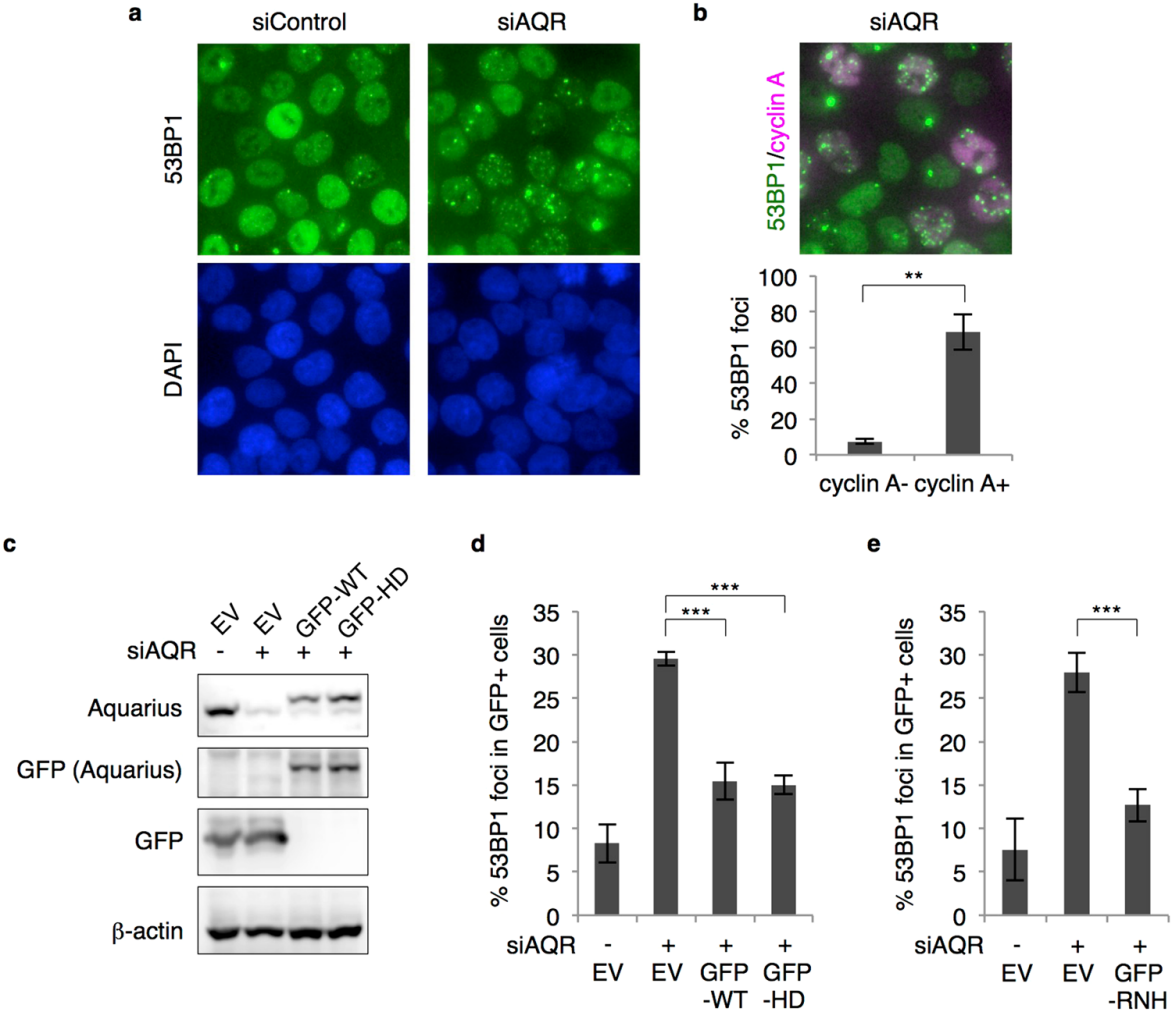
R-loop-mediated DNA damage accumulates in Aquarius-depleted cells. (**a**) Representative images of 53BP1 foci formation in *AQR*-depleted cells. HCT116 cells were transfected with *AQR* or control siRNA, and immunostained 48 h later with anti-53BP1 antibody. (**b**) siAQR-induced 53BP1 foci formation in cyclin A-positive cells. At 48 h after *AQR* siRNA transfection, HCT116 cells were stained with anti-53BP1 and anti-cyclin A antibodies. The percentages of 53BP1 foci (>5)-positive cells in cyclin A-negative and -positive cells were determined. Data represent mean ± SD from three independent experiments. (**c**) Expression of GFP-tagged Aquarius (WT and HD). HCT116 cells were transfected with GFP-Aquarius (WT and HD) or the empty vector (EV) and then transfected with siRNA 24 h after plasmid transfection. Cells were lysed 48 h later with SDS sample buffer, and expression of the indicated proteins was analysed by western blotting. (**d**) Percentage of 53BP1 foci (>5)-positive cells in *AQR*-depleted and -complemented cells shown in (**c**). Data represent mean ± SD from three independent experiments. (**e**) Percentage of 53BP1 foci (>5)-positive cells in GFP-RNase H1-expressing siAQR cells. HCT116 cells were transfected with GFP-RNase H1 plasmid or the empty vector (EV), followed by siRNA transfection. At 48 h after siRNA transfection, cells were immunostained with anti-53BP1 antibody and the percentage of 53BP1 foci-positive cells was determined. Data represent mean ± SD from three independent experiments. **p < 0.01; ***p < 0.005.

In Aquarius-depleted cells, spontaneous nuclear foci of γH2AX, a marker of DNA damage, emerge and DNA double-strand breaks accumulate^21^. This suggests that Aquarius suppresses genomic instability by resolving R-loops that can cause DNA damage. Alternatively, Aquarius may be involved in a pathway to repair DNA damage caused by the collision between DNA replication and R-loops. To test these hypotheses, we first analysed DNA damage caused by Aquarius depletion. *AQR*-knockdown cells showed spontaneous foci of 53BP1, a representative DNA damage response protein, in the absence of DNA-damaging agents (Fig. 1a), in agreement with a previous report^21^. These 53BP1 foci were mainly observed in cyclin A-positive cells, suggesting that the underlying damage arises through DNA replication (Fig. 1b). This 53BP1 foci formation was suppressed by expression of siRNA-resistant GFP-tagged wild-type (WT) Aquarius and a helicase-dead (HD) mutant (Y1196A)^22^ (Fig. 1c,d), as well as by GFP-RNase H1 overproduction (Fig. 1e). These results suggest that spontaneous DNA damage accumulates through collisions with DNA replication forks and R-loops in Aquarius-depleted cells. This R-loop formation and subsequent DNA damage were considered to be derived from the failure of splicing because Aquarius is a pre-mRNA splicing factor, but, interestingly, Aquarius helicase activity was not necessary to prevent the accumulation of spontaneous DNA damage. Thus, Aquarius helicase activity may be dispensable for pre-mRNA splicing.

### Aquarius contributes to the HR pathway

**Figure 2:**
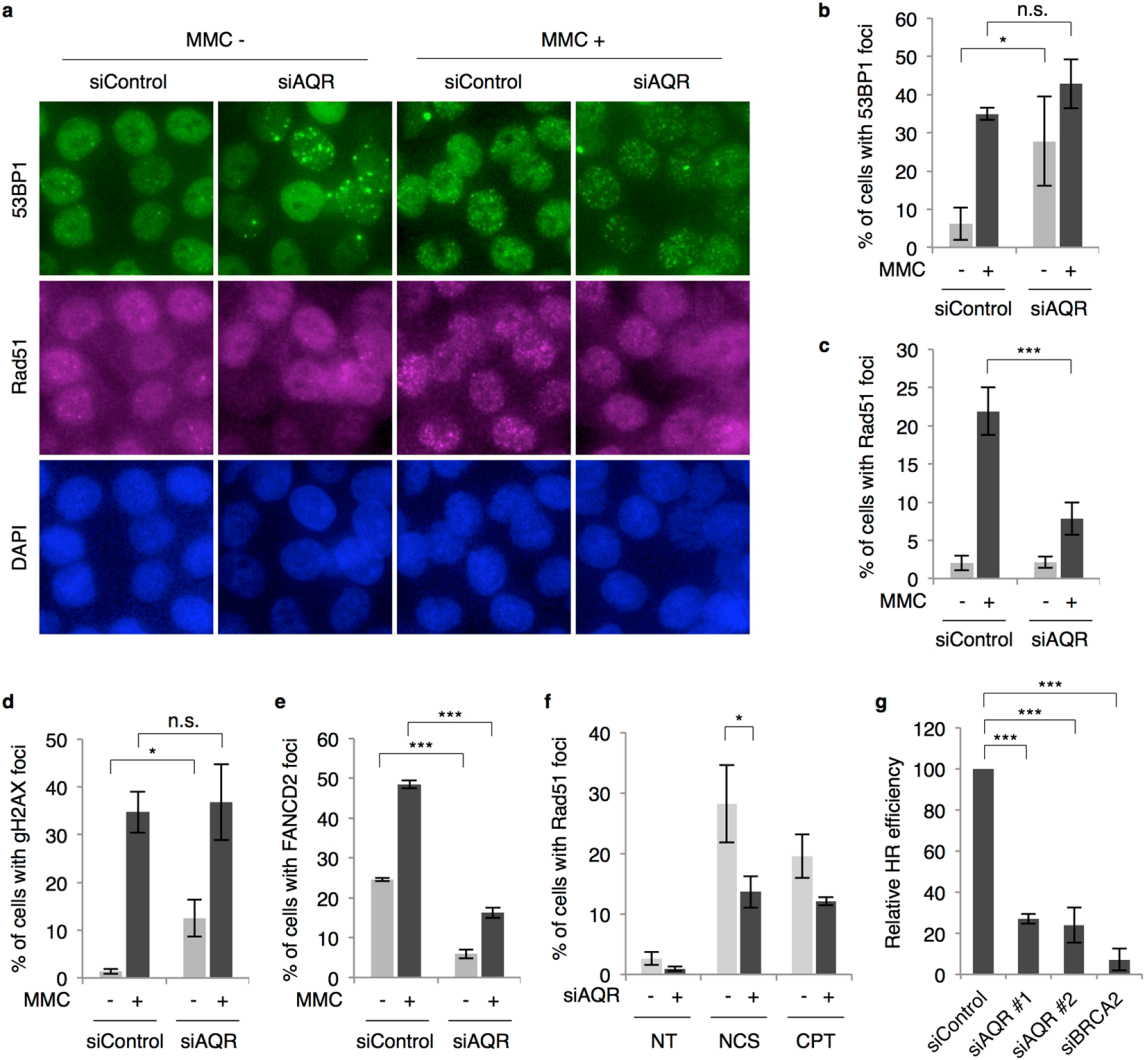
*AQR* knockdown suppresses HR. (**a**) Representative images of 53BP1 and Rad51 foci formation in *AQR*-knockdown cells. HCT116 cells were treated with or without MMC (200 ng/ml, 4 h) 48 h after siRNA transfection, and immunostained with anti-53BP1 or anti-Rad51 antibody. (**b**) Percentage of 53BP1 foci (>5)-positive cells in *AQR*-knockdown cells shown in (**a**). Data represent mean ± SD from three independent experiments. (**c**) Percentage of Rad51 foci (>10)-positive cells in *AQR*-knockdown cells shown in (**a**). (**d** and **e**) Percentage of γH2AX foci (>10)- and FANCD2 foci (>10)-positive cells in *AQR*-knockdown cells in response to MMC (200 ng/ml, 4 h). (f) Percentage of Rad51 foci-positive cells in *AQR*-knockdown cells in response to NCS (20 ng/ml, 4 h) or CPT (0.5 μM, 4 h). NT, no treatment. (**g**) DR-GFP assay for measuring HR frequency. DR-GFP U2OS cells were transfected with siRNA and then transfected with I-SceI expression plasmid 24 h after siRNA transfection. Percentages of GFP-positive cells were measured by flow cytometry. *p < 0.05; ***p < 0.005. n.s., not significant.

We next investigated cellular responses to DNA-damaging agents. We used mitomycin C (MMC), which causes DNA interstrand cross-links (ICLs) leading to replication stress and DNA strand breaks. In response to MMC, 53BP1 foci formation was comparable in *AQR*-knockdown cells and control cells (Fig. 2a,b). In contrast to 53BP1 foci formation, Aquarius depletion did not elevate Rad51 foci formation without MMC, and MMC-induced Rad51 foci formation was markedly suppressed in *AQR*-knockdown cells (Fig. 2a,c). Similar results were obtained using another siRNA targeting *AQR* (Supplementary Figure S1a), and Rad51 expression level was not changed by Aquarius depletion (Supplementary Figure S1b). To confirm this result, γH2AX and FANCD2 foci formation were additionally analysed. γH2AX is a phosphorylated form of histone H2AX that appears very early during DDR^26^, while FANCD2 is a key protein of the Fanconi anaemia pathway, which is correlated with HR derived from replication stress^27^. γH2AX foci formation showed a pattern similar to 53BP1 foci formation, whereas FANCD2 foci formation decreased, as in the case of Rad51 foci (Fig. 2d,e). Similar results were observed in cisplatin-treated cells (Supplementary Figure S1c). These findings suggest that Aquarius is involved in the HR pathway. However, DNA synthesis was partially downregulated in *AQR*-knockdown cells (Supplementary Figure S1d). To avoid possible cell cycle bias, we analysed Rad51 foci in cells expressing cyclin A, a marker of S and G2/M phases. Even in cyclin A-positive cells, Rad51 foci formation was suppressed by *AQR* knockdown (Supplementary Figure S1e). To see whether Aquarius is generally required for HR, cells were treated with neocarzinostatin (NCS) and camptothecin (CPT), two DNA-damaging agents that cause DSBs. *AQR* knockdown also suppressed Rad51 foci formation in response to DSBs as well as ICLs (Fig. 2f). These results indicate that Aquarius is involved in the HR pathway regardless of the cause of DSBs, but not in the early steps of DDRs, since damage-induced γH2AX and 53BP1 foci formation was not affected by Aquarius depletion. To confirm the contribution of Aquarius to HR, a DR-GFP assay was performed. HR efficiency decreased in *AQR*-knockdown cells as well as in positive control *BRCA2*-knockdown cells (Fig. 2g).

**Figure 3:**
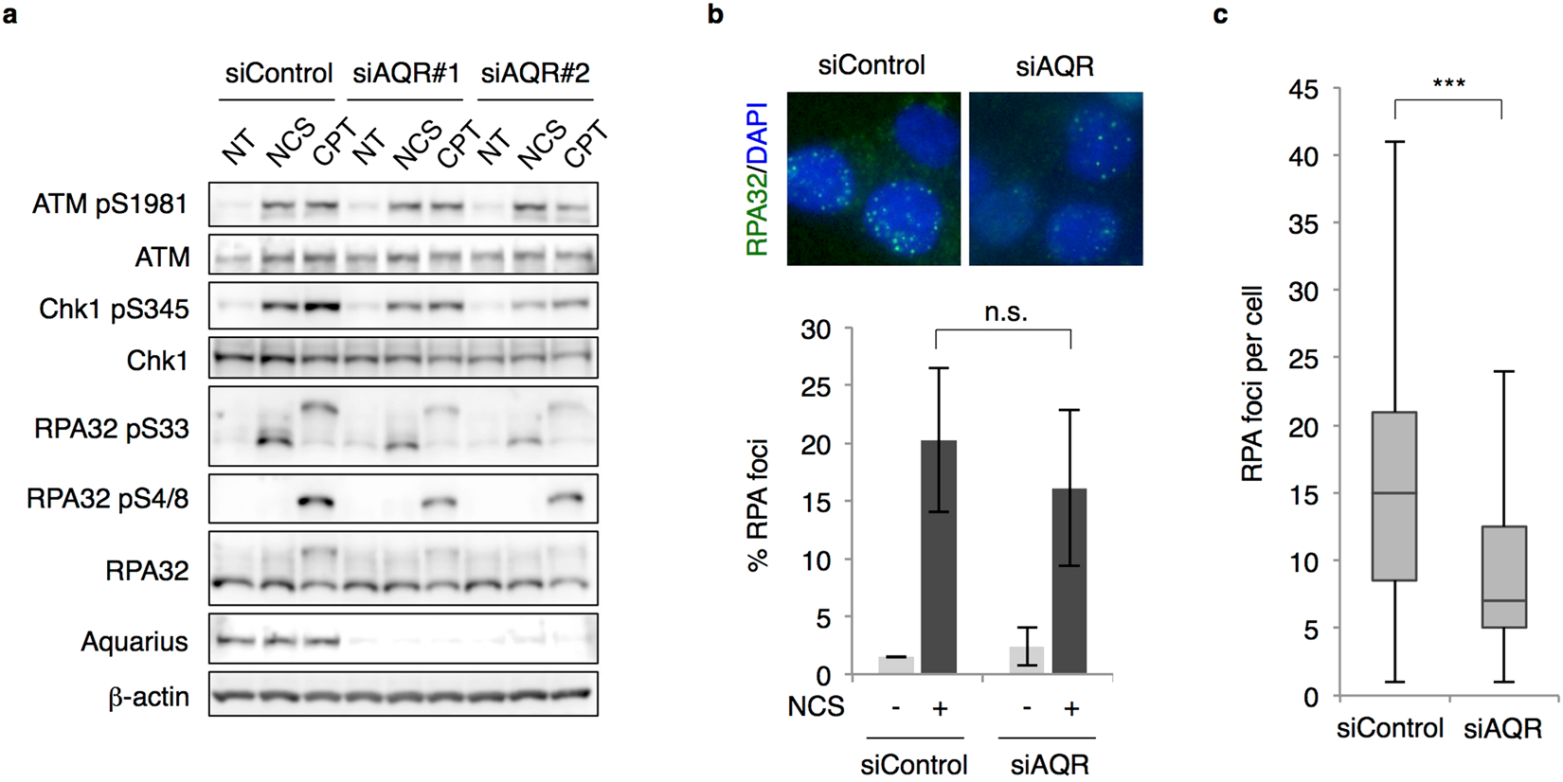
RPA loading is partially abrogated by *AQR* knockdown. (**a**) DSB responses in *AQR*-knockdown cells. U2OS cells were treated with NCS (20 ng/ml, 4 h) or CPT (1 μM, 2 h) 48 h after siRNA transfection. Cells were lysed with SDS sample buffer and the indicated proteins were detected by western blotting. NT, no treatment. (**b**) Percentage of RPA foci (>5)-positive cells. HCT116 cells were treated with NCS (20 ng/ml, 4 h) 48 h after siRNA transfection. Cells were pre-extracted with 0.2% Triton X-100/PBS prior to fixation and immunostained with anti-RPA32 antibody. Data represent mean ± SD from three independent experiments. n.s., not significant. (**c**) Number of RPA foci per cell. After RPA immunostaining, shown in (**b**), the numbers of RPA foci were counted in at least 60 cells and are shown as a boxplot. ***p < 0.005. n.s., not significant.

DNA end resection is required at the initiation of HR to generate a ssDNA region for strand exchange. To determine the effects of Aquarius on DNA end resection, DNA damage signalling, which is triggered by ssDNA formation, was analysed by immunoblotting. In response to DSBs, ATM was activated similarly in control and Aquarius-depleted cells, as assessed by autophosphorylation on Ser1981. On the other hand, Chk1 phosphorylation and RPA32 phosphorylation (Ser4/8, Ser33), representative of the DDR following DNA end resection, were partially suppressed by *AQR* knockdown (Fig. 3a). Furthermore, we analysed RPA32 foci formation in response to NCS, and found that the percentages of RPA32 foci-positive cells were comparable in control and *AQR*-knockdown cells (Fig. 3b). However, the number of foci per cell in *AQR*-knockdown cells was lower than that in control cells (Fig. 3c). This result suggests either that the number of DSB sites receiving DNA end resection is lower in Aquarius-depleted cells than in control cells, or that Aquarius is required for efficient loading of RPA proteins onto ssDNA regions.

### CtIP protein level is downregulated in Aquarius-depleted cells

**Figure 4:**
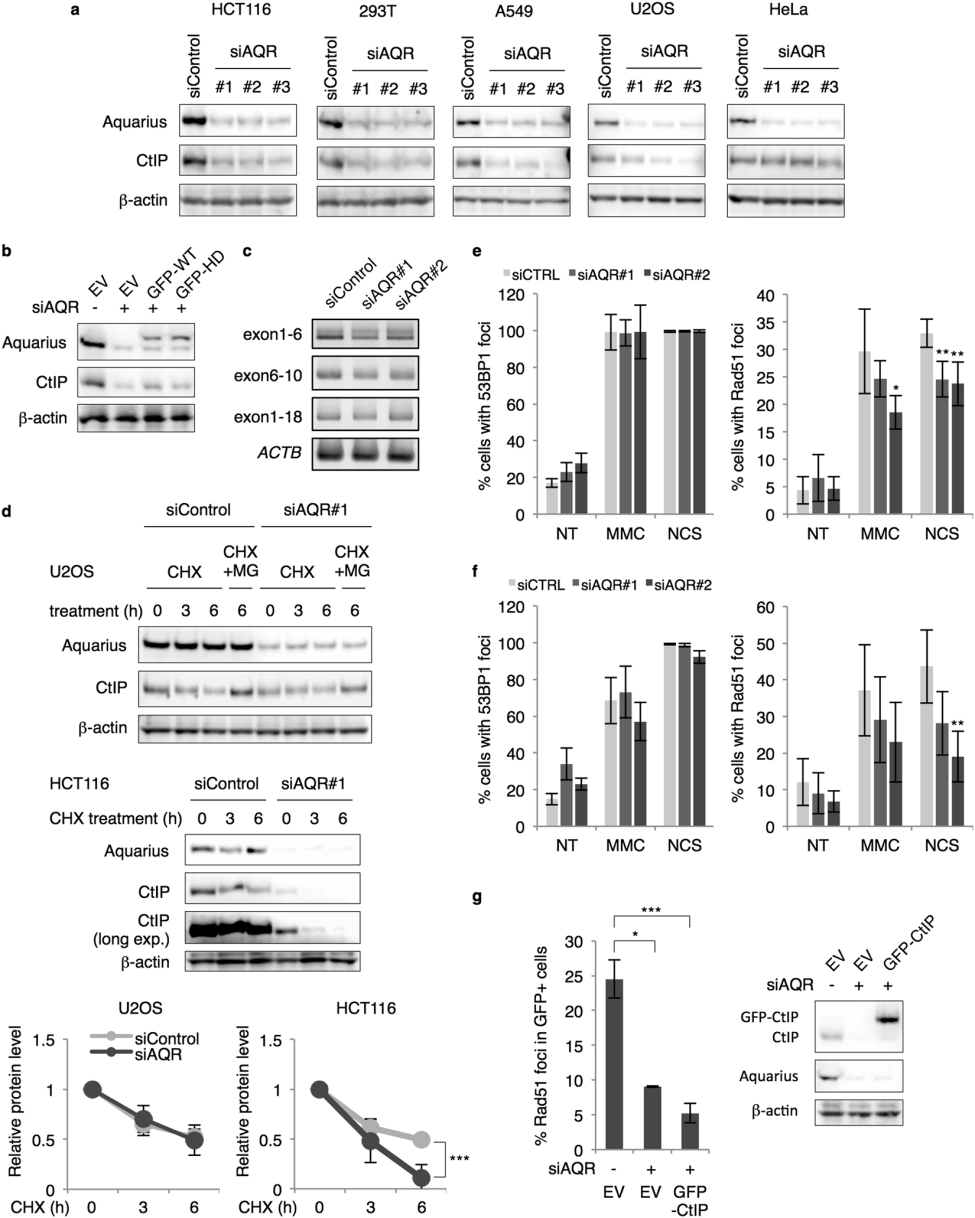
CtIP protein level is reduced in Aquarius-depleted cells. (**a**) Downregulation of CtIP protein level in *AQR*-knockdown cells. siRNAs against *AQR* were transfected into five different cell lines, and the cells were then lysed and CtIP and Aquarius were analysed by western blotting 48 h after siRNA transfection. (**b**) Partial rescue of CtIP by *AQR* add-back. Aquarius-depleted HCT116 cells transiently expressed GFP (EV) or GFP-tagged Aquarius (WT or HD), and CtIP protein was analysed by western blotting. (**c**) Analysis of CtIP mRNA by RT-PCR. Total RNA was isolated from HCT116 cells transfected with control siRNA or *AQR* siRNA, and PCR was performed with appropriate sets of primers after reverse transcription. RT-PCR against β-actin (*ACTB*) was performed as a loading control. (**d**) Protein stability of CtIP in *AQR*-knockdown cells. U2OS and HCT116 cells were treated with CHX (20 μg/ml) and MG-132 (MG, 10 μM) for the indicated periods 48 h after control siRNA or *AQR* siRNA transfection. Aquarius and CtIP protein levels were analysed by western blotting. Protein levels of CtIP were normalized by β-actin and the relative protein levels of CtIP are shown as a line graph. Data represent mean ± SD from at least three independent experiments. (**e** and **f**) Percentage of 53BP1 foci (>5)- and Rad51 foci (>10)-positive cells in *AQR*-knockdown HeLa (**e**) and U2OS (**f**) cells in response to MMC (200 ng/ml, 4 h) or NCS (20 ng/ml, 4 h). (**g**) Percentage of Rad51 foci (>10)-positive cells in CtIP overproduced *AQR*-knockdown cells. HCT116 cells, depleted for Aquarius, transiently expressed GFP (EV) or GFP-tagged CtIP (GFP-CtIP), and were immunostained with anti-Rad51 antibody after NCS (20 ng/ml, 4 h) treatment. Data represent mean ± SD from three independent experiments. *p < 0.05; **p < 0.01; ***p < 0.005.

To further investigate the role of Aquarius in RPA foci formation, we analysed CtIP, a factor responsible for DNA end resection, and surprisingly found that CtIP protein level was decreased in Aquarius-depleted cells (Fig. 4a). Among five different cell lines, HCT116, 293 T and A549 cells showed a significant decrease in CtIP protein level, whereas U2OS and HeLa cells showed a partial and no decrease, respectively. Expression of WT- and HD-Aquarius partially restored CtIP protein level (Fig. 4b), raising the possibility that the failure of RNA processing by Aquarius depletion causes the reduction of CtIP expression. We therefore checked the levels of CtIP mRNA by RT-PCR. Three sets of primers were used for RT-PCR to detect splicing abnormalities, but there were no notable differences in CtIP mRNA levels between control and *AQR*-knockdown cells (Fig. 4c). Next, to examine the stability of CtIP protein in *AQR*-knockdown cells, cells were treated with cycloheximide (CHX) and temporal changes of CtIP protein level were analysed. In U2OS cells, which showed a partial reduction of CtIP, comparable decreases of CtIP protein following CHX treatment were observed in both control and *AQR*-knockdown cells (Fig. 4d). In contrast to U2OS cells, in HCT116 cells, which showed a drastic reduction of CtIP, a marked decrease of CtIP protein after CHX treatment was observed in *AQR*-knockdown cells in comparison to control cells. These results imply that CtIP reduction in Aquarius-depleted cells is due to destabilization of the protein.

This finding suggested to us that the HR defect observed in *AQR*-knockdown cells is the consequence of CtIP reduction. To examine this possibility, we further analysed Rad51 foci formation in U2OS and HeLa cells, both of which show minor effects on CtIP protein level in response to *AQR* knockdown. U2OS and HeLa cells showed a significant decrease of Rad51 foci formation in response to NCS, but the degree of suppression of Rad51 foci formation in both cells was smaller than that in HCT116 cells (Fig. 4e,f). Moreover, overproduction of GFP-CtIP in *AQR*-knockdown HCT116 cells did not rescue Rad51 foci formation (Fig. 4g). These data suggest that CtIP protein reduction is not sufficient to explain the suppressive effect on HR of Aquarius depletion, and that Aquarius has another role in HR in addition to its contribution to CtIP protein stabilization. The DR-GFP assay with U2OS cells shown in Fig. 2g also supports this conclusion. Furthermore, the suppression of FANCD2 foci formation in *AQR*-knockdown cells shown in Fig. 2e suggests a role for Aquarius in HR other than CtIP downregulation, because CtIP downregulation does not reduce FANCD2 foci formation^28^.

### Aquarius may contribute to HR through R-loop resolution

**Figure 5:**
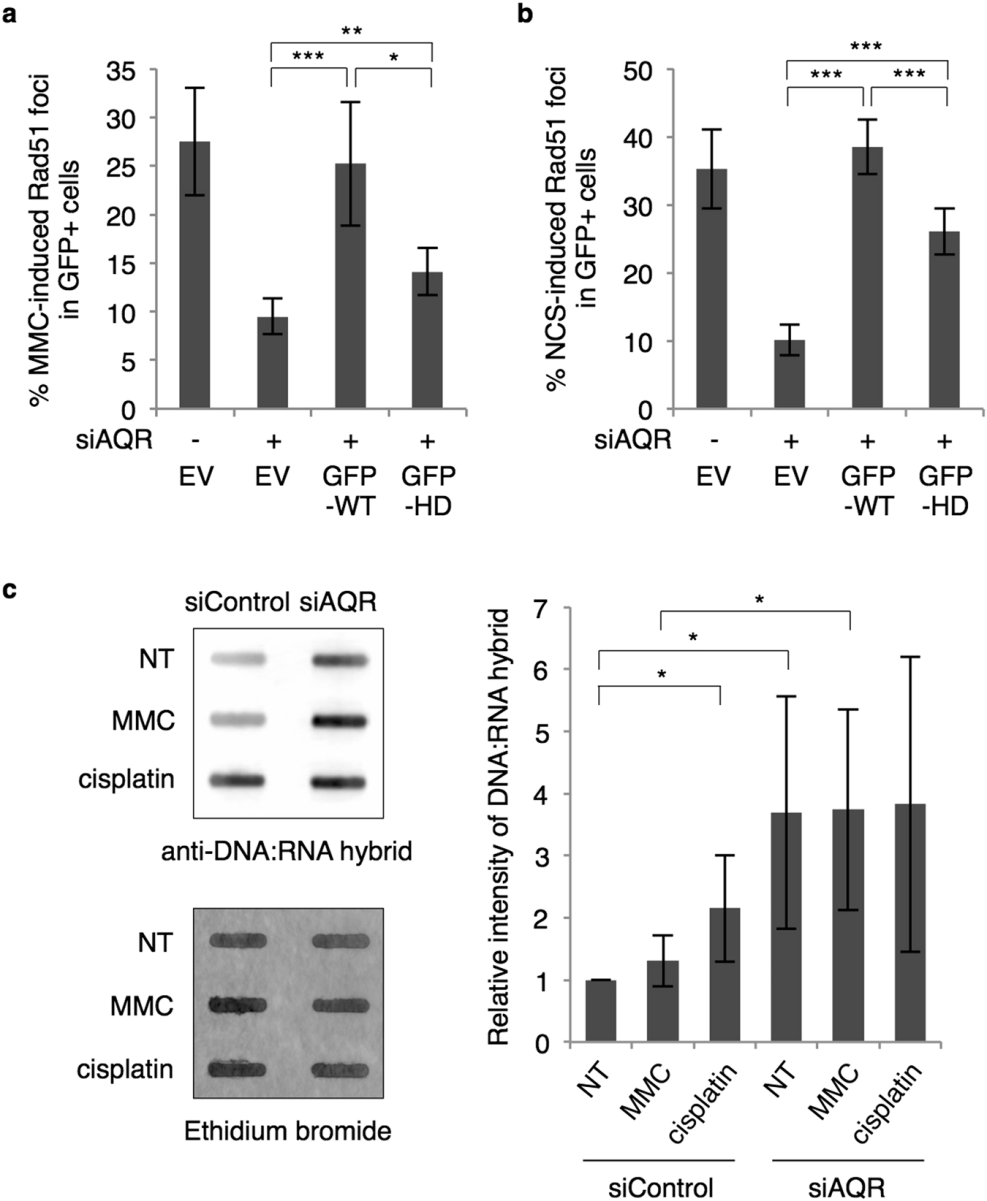
Aquarius helicase activity is required for HR promotion. (**a** and **b**) Percentage of Rad51 foci-positive cells in *AQR*-knockdown or *AQR* (WT or HD)-complemented cells. HCT116 cells, depleted for Aquarius, transiently expressed GFP (EV) or GFP-tagged Aquarius (WT or HD), and were immunostained with anti-Rad51 antibody after MMC (**b**, 200 ng/ml, 4 h) or NCS (**c**, 20 ng/ml, 4 h) treatment. Data represent mean ± SD from four (**a**) or five (**b**) independent experiments. (**c**) Analysis of R-loop accumulation in *AQR*-depleted cells. At 48 h after siRNA transfection of HCT116, cells were treated with MMC or cisplatin and genomic DNA was then isolated. R-loop formation was detected by slot blotting with anti-DNA-RNA hybrid antibody. The relative values normalized by ethidium bromide intensity are shown to the right. NT, no treatment. Data represent mean ± SD from five independent experiments. *p < 0.05; **p < 0.01; ***p < 0.005.

Since Aquarius is an RNA helicase and resolves R-loops, we hypothesized that Aquarius helicase activity is required for HR. To test this hypothesis, we transiently transfected GFP-WT- or GFP-HD-Aquarius into HCT116 cells depleted of endogenous Aquarius. MMC- and NCS-induced Rad51 foci formation in GFP-positive cells were fully restored by expression of WT-Aquarius, whereas the restoration by HD-Aquarius expression was partial (Fig. 5a,b). These data indicate that Aquarius helicase activity is required for efficient Rad51 foci formation. Because Aquarius helicase activity is considered to be involved in R-loop resolution, we next determined whether R-loops accumulate in *AQR*-knockdown cells. Genomic DNA was isolated and DNA-RNA hybrids were directly detected by slot blot using an anti-DNA-RNA hybrid antibody, S9.6. Confirming a previous report^21^, DNA-RNA hybrids accumulated highly in *AQR*-knockdown cells, in contrast to control cells (Fig. 5c). Interestingly, MMC treatment tended to increase R-loops, and cisplatin treatment significantly increased R-loops. These results raise the possibility that DNA damage generated in transcriptionally active regions induces the formation of R-loops around damage sites, whereupon Aquarius removes RNA from R-loops to promote Rad51 loading.

In addition to Aquarius, several other proteins involved in R-loop processing have been analysed for their role in DDRs. In *XPF*-knockdown or -deficient cells, there were no significant differences in MMC-induced 53BP1 and Rad51 foci formation (Supplementary Figure S2a, S2b, and S2c). Similarly, knockdown of *CSB* did not affect either 53BP1 or Rad51 foci formation in our hands (Supplementary Figure S2d and S2e). Although this result is not consistent with a recent report that CSB knockout diminishes HR^29^, this inconsistency may be due to the different methods used to deplete CSB or to induce DNA damage. Depletion of senataxin (*SETX*), an R-loop processing helicase, also had no significant effect on 53BP1 or Rad51 foci formation in response to MMC (Supplementary Figure S2f and S2g).

Aquarius forms splicing complexes with several proteins including XAB2 and CCDC16^22,24^. *XAB2* knockdown, but not *CCDC16* knockdown, yielded numerous spontaneous foci of 53BP1 and fewer damage-induced Rad51 foci (Supplementary Figure S3a). Aquarius may function in different aspects of RNA metabolism depending on each complex in which it participates. Aquarius protein level was reduced by *XAB2* knockdown, in agreement with a previous report (Supplementary Figure S3b)^24^, whereas *AQR* knockdown did not affect XAB2 level (Supplementary Figure S1b). Aquarius may be stabilized by binding to XAB2 and may function in HR as a complex with XAB2 in response to DNA damage. Indeed, XAB2 has been reported to contribute to HR^30^. However, this contribution remains uncertain because *XAB2* knockdown causes severe growth defects and a partial reduction of Rad51 protein level (Supplementary Figure S3b).

### Aquarius is required for cell survival against genotoxic agents

**Figure 6:**
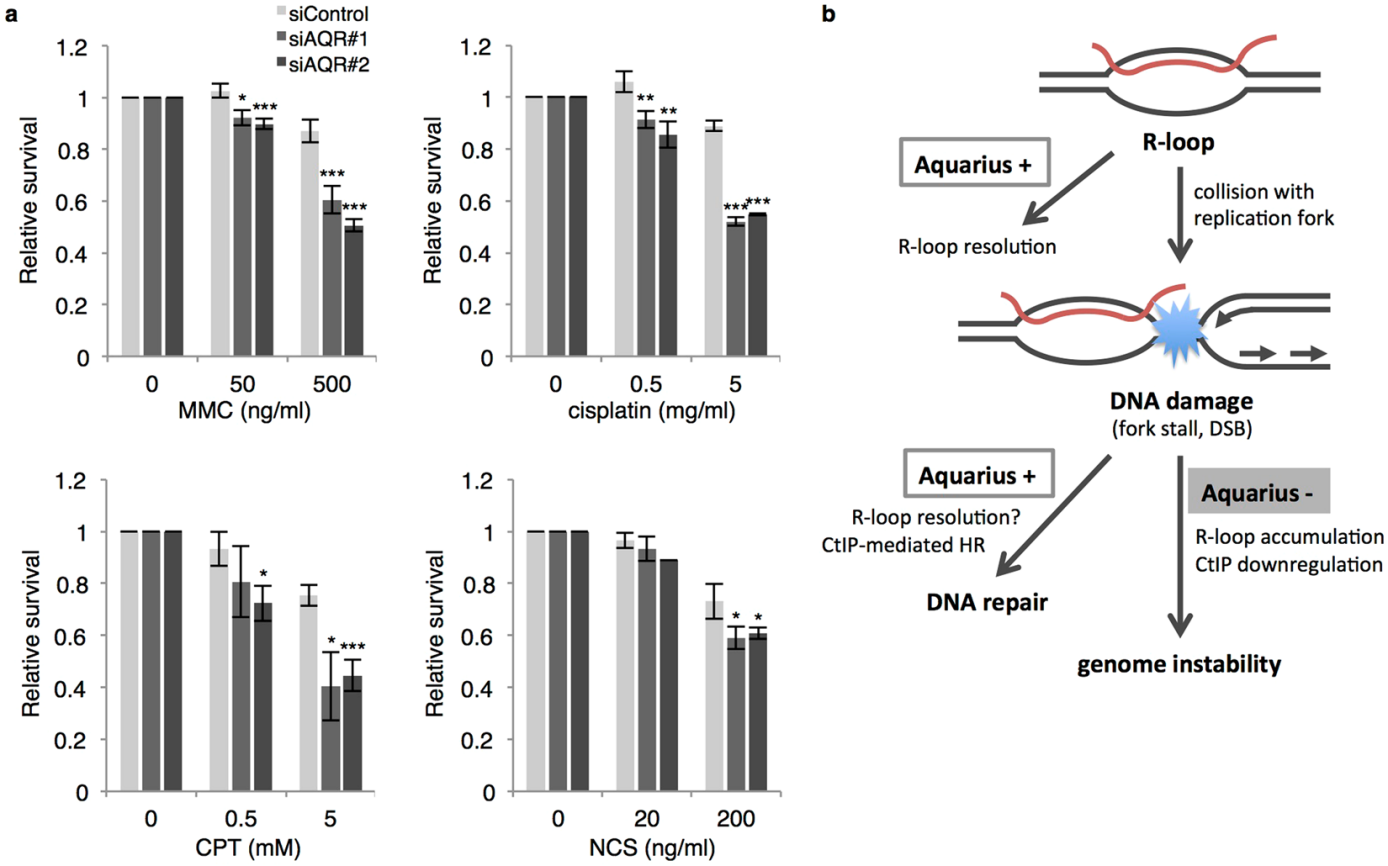
Aquarius depletion sensitizes cells to genotoxic agents. (**a**) Cell survival assay in *AQR*-knockdown cells treated with genotoxic agents. After transfection with the indicated siRNAs, HCT116 cells were plated into a 96-well plate. Cell survival was assayed using a Cell Titer-Glo kit as described in Materials and Methods. Data represent mean ± SD from three independent experiments. *p < 0.05; **p < 0.01; ***p < 0.005. (**b**) Schematic model of how Aquarius contributes to the maintenance of genomic integrity. Spontaneous R-loops are resolved by Aquarius, or else they collide with DNA replication resulting in replication fork stall or DSB generation. Meanwhile, collision between DNA damage and transcription results in R-loop formation around DNA damage sites. In the presence of Aquarius, R-loops may be removed from DNA damage sites by Aquarius and subsequently DNA damage is repaired by the HR pathway. On the other hand, Aquarius depletion reduces HR efficiency, probably by CtIP downregulation and R-loop persistence.

Given that Aquarius contributes to HR, we presumed that Aquarius-depleted cells would be sensitive to DNA-damaging agents. Thus, we performed a cell survival assay using several DNA-damaging agents. As expected, *AQR*-knockdown cells were more sensitive to MMC and cisplatin, and CPT also induced more cell death in *AQR*-knockdown cells, compared to control cells. Following NCS treatment, *AQR*-knockdown cells were significantly more sensitive than control cells (Fig. 6a).

## Discussion

R-loops are naturally generated in some cases of RNA metabolism, and a resultant R-loop can collide with a DNA replication fork. Such collisions result in replication fork stalling and DSB generation^31,32^. In another case, DNA damage-sensing kinases including ATM and DNA-PK are activated in response to DSBs and repress transcription around DSB sites^33–36^. These reports prompted us to hypothesize that transcription machineries are stalled around DSB sites in a transcriptionally active region, after which RNA polymerases may become detached from DNA and the transcribed RNA may remain to form an R-loop. In accordance with these possibilities, RNase H1 is recruited to sites of DNA damage caused by micro-laser irradiation, suggesting R-loop formation at the DNA damage sites^37,38^. A more recent paper has directly shown that DNA-RNA hybrids and RNA polymerase II accumulate around DSB sites in yeast cells^39^.

Since Aquarius is a splicing factor, the spontaneous 53BP1 foci observed in Aquarius-depleted cells are most likely to be derived from splicing failure. A previous study indicated that Aquarius is required for *in vitro* assembly of the splicing complex, but that its helicase activity is not required^22^, suggesting a model in which Aquarius solves abnormal base pairing and displaces proteins from mRNA to promote proper folding of mRNA. According to this model, the splicing machinery is stalled in the absence of Aquarius, which may cause formation of R-loops and subsequently DNA damage, resulting in 53BP1 foci formation. Consistent with this idea, we found that DNA damage caused by Aquarius depletion was cancelled by RNase H1 overproduction. However, HD-Aquarius was able to rescue the spontaneous DNA damage, implying that Aquarius helicase activity is dispensable for the progression of pre-mRNA splicing. In addition to this role of Aquarius to protect cells from spontaneous DNA damage, we propose a new role for Aquarius in the maintenance of genomic stability. Aquarius depletion abrogated HR and sensitized cells to DNA-damaging agents. In addition, we unexpectedly found a downregulation of CtIP protein level in *AQR*-knockdown cells, which immediately suggested a correlation with the HR defect in *AQR*-knockdown cells. The mechanism of Aquarius-dependent CtIP stabilization is a prominent remaining question. Aquarius may form a complex with and stabilize CtIP, but we have not yet detected a clear interaction between Aquarius and CtIP. Also in XAB2-depleted cells, CtIP protein level was reduced as well as Aquarius protein level (Supplementary Figure S3c). This may reflect that XAB2 acts as a core factor to stabilize a heterotrimeric complex including Aquarius and CtIP. However, Onyango *et al*. have reported no effect of XAB2 depletion on CtIP protein level or DNA end resection^30^. The effect of XAB2 on the Aquarius complex is still controversial and needs further analysis to be revealed in the future.

Although CtIP was an evident candidate to elucidate the role of Aquarius on HR, exogenous CtIP expression did not rescue the HR defect in *AQR*-knockdown cells. Moreover, HD-Aquarius was unable to fully rescue damage-induced Rad51 foci formation. These data cannot explain the phenotypes of *AQR*-knockdown cells, and clearly indicate that Aquarius helicase activity is required for promotion of HR in addition to CtIP stabilization, probably through RNA removal from R-loops accumulated around damage sites (Fig. 6b). However, the incomplete rescue by add-back of HD-Aquarius may imply that other helicases or nucleases potentially forming a complex with Aquarius contribute to RNA removal. One such helicase may be DDX1, which has been reported to contribute to RNA removal around DSB caused by I-SceI endonuclease^40^.

The biggest remaining challenge is direct detection of R-loops or RNA molecules and Aquarius at natural DNA damage sites. Information about Aquarius localization during DDRs is still lacking, because Aquarius did not show typical damage-induced nuclear foci in our experiments. We think that one or a few molecules of RNA and Aquarius are involved in HR at DSB sites associated with R-loop formation. Further elucidation of these questions will require a new method involving high-resolution microscopy and R-loop capture. In the International Cancer Genome Consortium database, numerous mutations in the *AQR* gene in various cancers are accumulating (https://dcc.icgc.org), raising the possibility that Aquarius functions in the maintenance of genome integrity to prevent carcinogenesis, and that further studies on RNA biogenesis factors including Aquarius will improve our understanding of the mechanisms of genome stability and stimulate the development of new cancer therapies.

## Methods

### Cell culture and reagents

HCT116, U2OS, A549 and 293 T cells were maintained in DMEM containing 10% foetal bovine serum. MMC and cisplatin were purchased from Nacalai Tesque and Nichi-Iko Pharmaceutical, respectively. NCS, CPT, CHX and MG-132 were purchased from Sigma-Aldrich.

### Immunostaining and western blotting

For immunostaining, cells were fixed with 4% paraformaldehyde and then permeabilized with PBS containing 0.5% Triton X-100. The fixed cells were stained with primary and secondary antibodies conjugated to Alexa488 or Alexa594, followed by counterstaining with 4, 6-diamino-2-phenylindole (DAPI) for the nucleus. The samples were visualized using an IX71 fluorescence microscope (Olympus). For western blotting, cells were lysed with SDS sample buffer and proteins were separated by SDS-polyacrylamide gel electrophoresis. After transfer to a PVDF membrane, proteins were detected with primary antibodies followed by secondary antibody conjugated with horseradish peroxidase. Chemiluminescent signals were detected using a C-Digit Blot Scanner (LI-COR). The primary antibodies used in this study were as follows: anti-53BP1 (Bethyl Laboratories, A300–272A), anti-Rad51 (kindly gifted by Dr. Akira Shinohara, Osaka University; Abnova, H00005888-B01P), anti-γH2AX (Merck Millipore, 05–636), anti-FANCD2 (Novus, NB100–182), anti-GFP (Santa Cruz Biotechnology, sc-9996), anti-ATM pS1981 (Epitomics, 2152-1), anti-ATM (Cell Signaling Technology, #2873), anti-Chk1 pS345 (Cell Signaling Technology, #2348), anti-Chk1 (Santa Cruz Biotechnology, sc-8408), anti-RPA32 (Abcam, ab2175), anti-RPA32 pS33 (Abcam, ab87278), anti-RPA32 pS4/8 (Bethyl Laboratories, A300-245A), anti-Aquarius (Bethyl Laboratories, A302-547A), anti-β-actin (Abcam, ab6276), anti-cyclin A (Santa Cruz Biotechnology, sc-751), anti-XPF (clone 19-16, originally generated in Dr. Tsukasa Matsunaga’s laboratory), anti-CSB (Santa Cruz Biotechnology, sc-25370), anti-senataxin (Novus, NBP1-94712), anti-XAB2 (Bethyl Laboratories, A303-637A), anti-CCDC16 (Bethyl Laboratories, A301-419A) and anti-CtIP (Cell Signaling Technology, #9201).

### Detecting R-loops by slot blot

To detect R-loop formation, we modified a previously published protocol^21^. Genomic DNA was isolated using a QIAamp-DNA-Blood kit (QIAGEN) following the manufacturer’s protocol. Genomic DNA (1 μg) was transferred to a positively charged nylon membrane using a slot blot apparatus, and then cross-linked by UV irradiation. After blocking with 5% skim milk, the membrane was incubated with anti-DNA-RNA hybrid antibody (S9.6, kindly gifted by Dr. Stephen Leppla, NIH) followed by secondary antibody-conjugated horseradish peroxidase. The chemiluminescent signals were measured using a C-Digit Blot Scanner. To normalize the DNA-RNA hybrid signal, the membrane was stained with ethidium bromide and DNA was detected by blue light using the Fusion system (Vilber Lourmat).

### RT-PCR

Total mRNA was isolated from cells transfected with siRNAs using an RNeasy Plus Mini Kit (QIAGEN), and a reverse transcription reaction was performed using an Omniscript RT Kit (QIAGEN) following the manufacturer’s protocol. PCR was performed with specific primers against the CtIP gene and EX Taq DNA polymerase (TAKARA BIO). Primer sequences were as follows: TGTGGAAGCCCTAACTCTGC and ACGCCAGAAAATGAGAAGGC for exon1–6; TGGAGCACTCTGTGTGTGC and GCATTCCTGTGAACAGGGC for exon6–10; and ATTGCGTTGTAAGGCTGACG as a reverse primer for exon1–18.

### Cell survival assay

After siRNA transfection, cells were plated into a clear-bottom 96-well plate and incubated for 48 h. The cells were treated with genotoxic agents for 6 h and cell survival was measured using a Cell Titer-Glo kit (Promega) 42 h after washing out of genotoxic agents.

### Gene knockdown and add-back experiments

Cells were transfected with siRNA by RNAiMAX (Invitrogen) following the manufacturer’s protocol. At 48 h after transfection, cells were treated with genotoxic agents for immunostaining assay or western blotting. siRNAs used in this study were as follows: AQR#1 (Ambion, s18725), AQR#2 (Ambion, s18726), SETX (CCAUCUAACUCUGUACAACUUGCUU^41^), CSB (CCACUGAUUACGAGAUACA^42^), XPF (Ambion, s4799), CCDC16#1 (Ambion, s40699), CCDC16#2 (Ambion, s40700), XAB2 (Ambion, s32465), BRCA2 (GAAGAAUGCAGGUUUAAUA) and control siRNA (Ambion Silencer Negative Control #1 siRNA or Sigma-Aldrich MISSION siRNA Negative Control #2). For Aquarius add-back experiments, *AQR* cDNA was cloned into the pEGFP-C1 vector. Cells were transfected with Aquarius expression plasmid or empty plasmid using FuGENE HD (Promega), and then transfected with siRNA using RNAiMAX 24 h after plasmid transfection. After a further 48 h, cells were treated with MMC or NCS and were analysed for Rad51 foci formation. For GFP-CtIP transfection, Viafect (Promega) was used.

### Measuring HR efficiency using the DR-GFP system

Direct repeat (DR)-GFP U2OS cells were transfected with siRNAs using HiPerFect transfection reagent (QIAGEN). After 24 h, cells were transfected again with siRNAs, and then with either EGFP-C1 or I-SceI vector, using Lipofectamine 3000 (Thermo Fisher Scientific), 24 h after the second siRNA transfection. Percentages of GFP-positive cells were measured using an Attune acoustic focusing cytometer (Thermo Fisher Scientific) at 48 h after the I-SceI transfection.

## Electronic supplementary material

Supplementary Figures
