# Supplementary material for: Aquarius is required for proper CtIP expression and homologous recombination repair
Source: Sci Rep. 2017 Oct 23;7:13808. doi: 10.1038/s41598-017-13695-4 (PMC5653829; doi:10.1038/s41598-017-13695-4)
Supplement: Supplementary file 1 — Supplementary Figures [file 41598_2017_13695_MOESM1_ESM.pdf]

Supplementary information

Aquarius is required for proper CtIP expression and homologous recombination repair

Ryo Sakasai[1], Mayu Isono[2], Mitsuo Wakasugi[3], Mitsumasa Hashimoto[4], Yumi Sunatani[1], Tadashi Matsui[1], Atsushi Shibata[2], Tsukasa Matsunaga[3], Kuniyoshi Iwabuchi[1*]

1, Department of Biochemistry I, Kanazawa Medical University
2, Education and Research Support Center, Gunma University
3, Faculty of Pharmacy, Institute of Medical, Pharmaceutical and Health Sciences, Kanazawa University
4, Department of Physics, Kanazawa Medical University

*Corresponding author

Supplementary Figure Legends

Figure S1. (a) DDRs in cells knocked down by #2 siRNA targeting *AQR*. HCT116 cells were transfected with control siRNA or #2 siRNA targeting *AQR*. At 48 h after transfection, cells were treated with MMC (200 ng/ml, 4 h). 53BP1 and Rad51 foci formation were analysed by immunostaining. (b) *AQR* knockdown efficiency. HCT116 cells were transfected with *AQR* siRNAs and the indicated proteins were analysed by western blotting with specific antibodies. (c) Cisplatin-induced Rad51 foci formation in *AQR*-knockdown cells. HCT116 cells transfected with *AQR* siRNA were treated with cisplatin (2.5 μg/ml, 4 h) and immunostained with anti-Rad51 antibody. (d) DNA synthesis in *AQR*-knockdown cells. At 48 h after siRNA transfection into HCT116 cells, newly synthesized DNA was pulse-labelled with EdU for 30 min and analysed by flow cytometry. (e) Rad51 foci formation in cyclin A-positive cells with or without *AQR* knockdown. At 48 h after siRNA transfection, HCT116 cells were treated with MMC (200 ng/ml, 4 h) and co-immunostained with anti-Rad51 and anti-cyclin A antibodies. Rad51 foci-positive cells were counted in cyclin A-positive cells. Data represent mean ± SD from three independent experiments.

Figure S2. (a and b) MMC-induced DDRs in *XPF*-knockdown cells. HCT116 cells transfected with control siRNA or *XPF* siRNA were treated with MMC (200 ng/ml, 4 h), and immunostained with anti-53BP1 and anti-Rad51 antibodies (a). Knockdown efficiency was assessed by western blotting with anti-XPF antibody (b). (c) Rad51 foci formation in XPF-deficient cells. Control cells (WI38VA13) and XPF-deficient cells (XP2YOSV) were treated with MMC (200 ng/ml, 4 h) and immunostained with anti-Rad51 antibody. (d and e) MMC-induced DDRs in *CSB*-knockdown cells. HCT116 cells transfected with control siRNA or *CSB* siRNA were treated with MMC (200 ng/ml, 4 h), and immunostained with anti-53BP1 and anti-Rad51 antibodies (d). Knockdown efficiency was assessed by western blotting with anti-CSB antibody (e). (f and g) MMC-induced DDRs in *SETX*-knockdown cells. HCT116 cells transfected with control siRNA or *SETX* siRNA were treated

with MMC (200 ng/ml, 4 h), and immunostained with anti-53BP1 and anti-Rad51 antibodies (f). Knockdown efficiency was assessed by western blotting with anti-senataxin antibody (g). n.s., not significant. Data represent mean ± SD from three independent experiments.

Figure S3. (a and b) DDRs in *XAB2*-knockdown cells. At 48 h after *XAB2* siRNA transfection, HCT116 cells were treated with MMC and immunostained with anti-53BP1 and anti-Rad51 antibodies (a). Knockdown efficiency was assessed by western blotting with specific antibodies against the indicated proteins (b). (c) CtIP protein level in *XAB2*-knockdown cells. After XAB2 siRNA transfection, CtIP was analysed by western blotting. (d and e) DDRs in *CCDC16*-knockdown cells. At 48 h after *CCDC16* siRNA transfection, HCT116 cells were treated with MMC and immunostained with anti-53BP1 and anti-Rad51 antibodies (d). Knockdown efficiency was assessed by western blotting with specific antibodies against the indicated proteins (e). Data represent mean ± SD from three independent experiments.

Figure S4. (a) Original data of Aquarius add-back experiment related to Figure 1c. (b) Original data of DDRs in *AQR*-knockdown cells related to Figure 3a.

Figure S5. (a) Original data of CtIP protein level in *AQR*-knockdown cells related to Figure 4a. (b) Original data of CtIP protein level in Aquarius-complemented cells related to Figure 4b. (c) Original data of CtIP stability in *AQR*-knockdown cells related to Figure 4d.

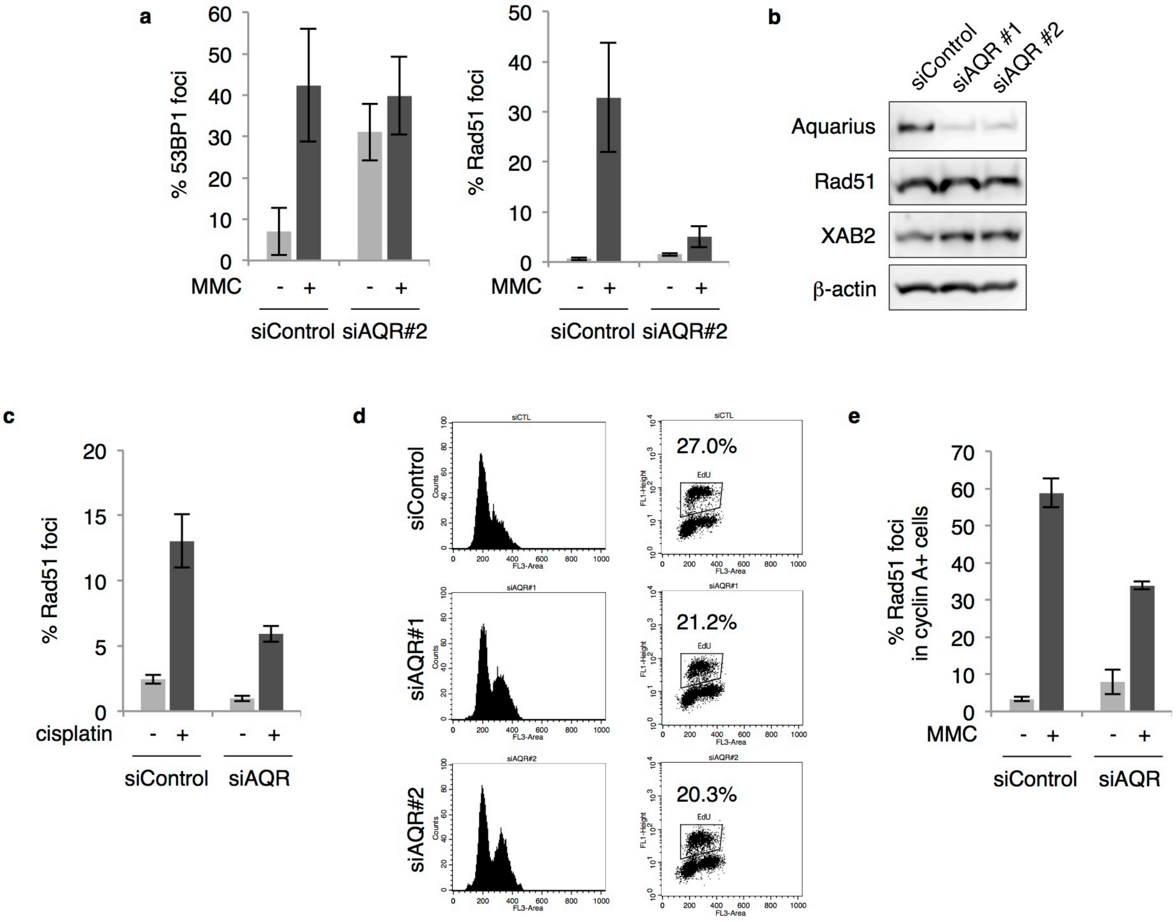

Figure S1
Sakasai et al. Aquarius is required for proper CtIP expression and homologous recombination repair.

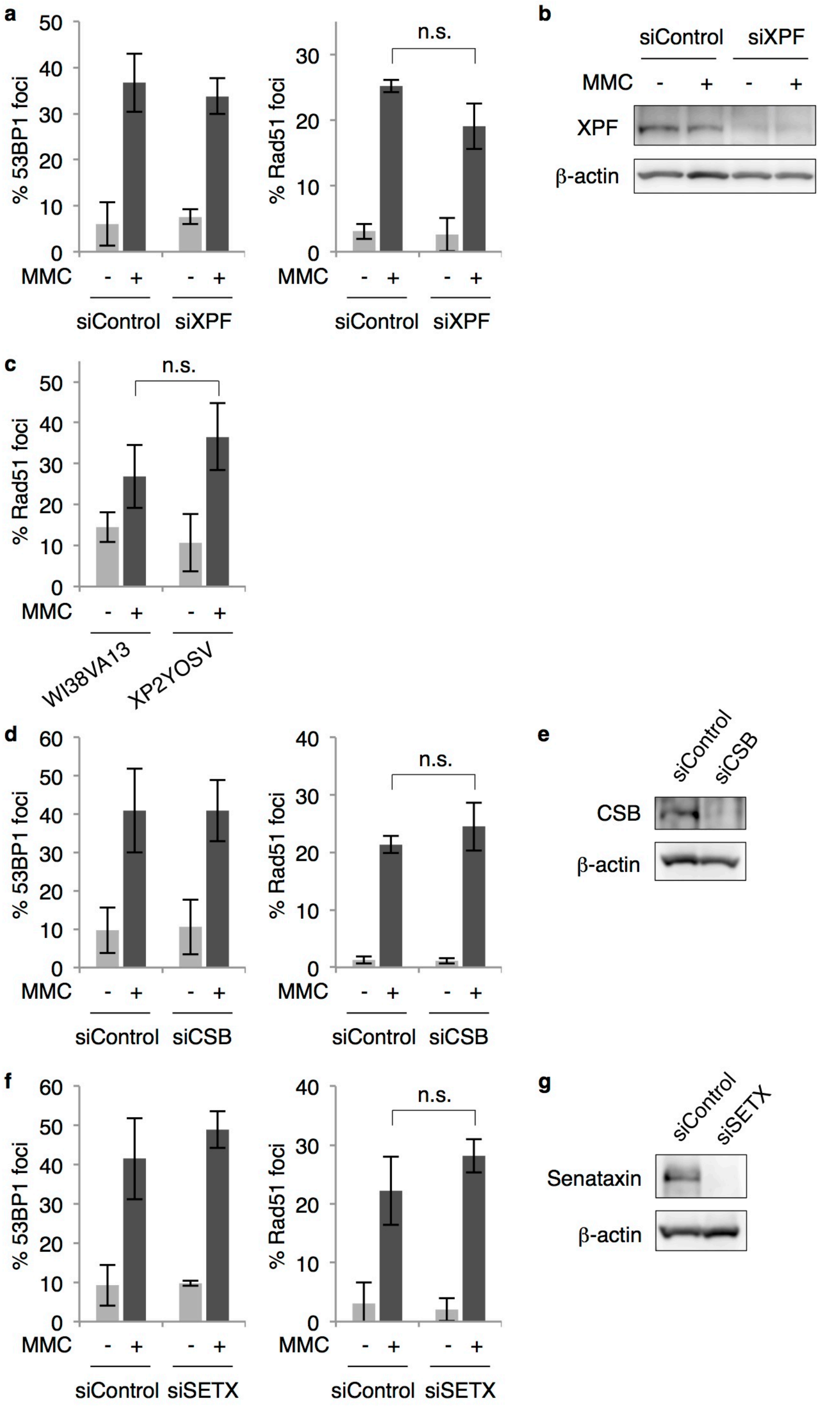

Figure S2

Sakasai et al. Aquarius is required for proper CtIP expression and homologous recombination repair.

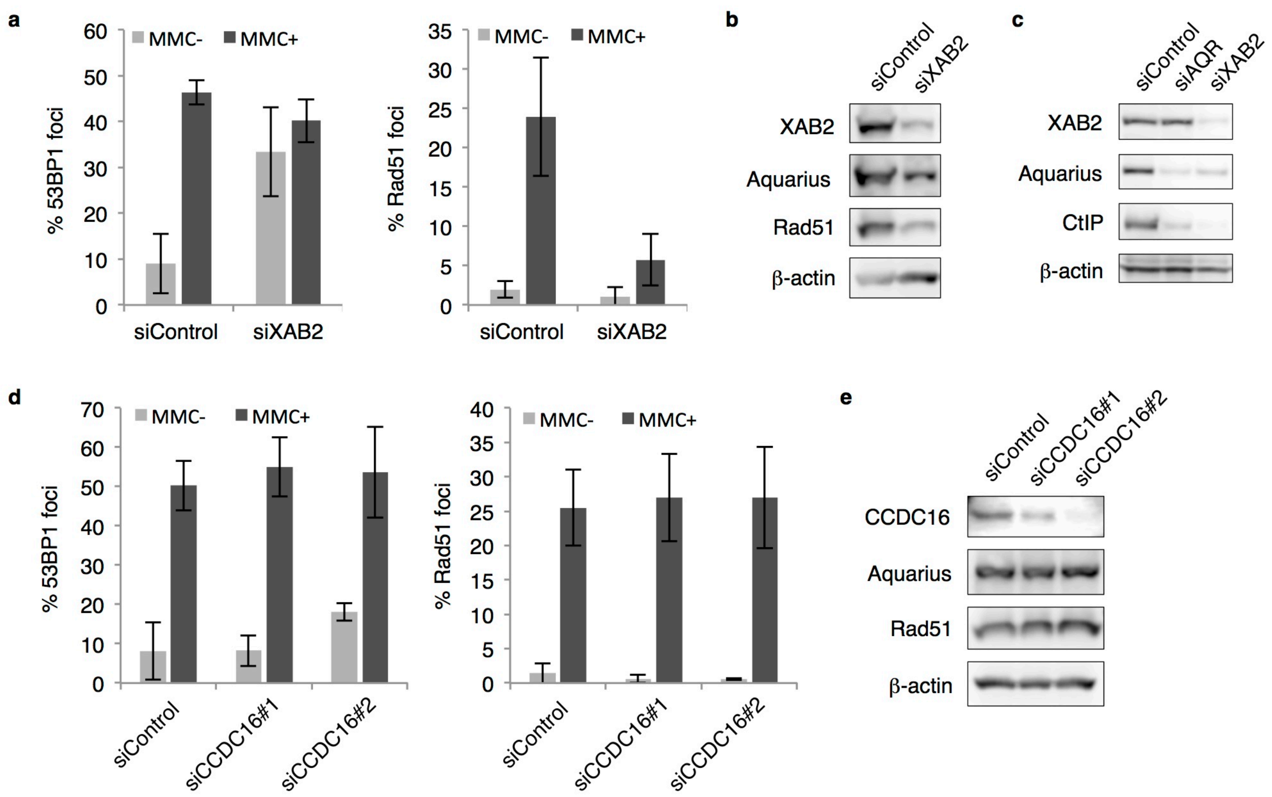

Figure S3
Sakasai et al. Aquarius is required for proper CtIP expression and homologous recombination repair.

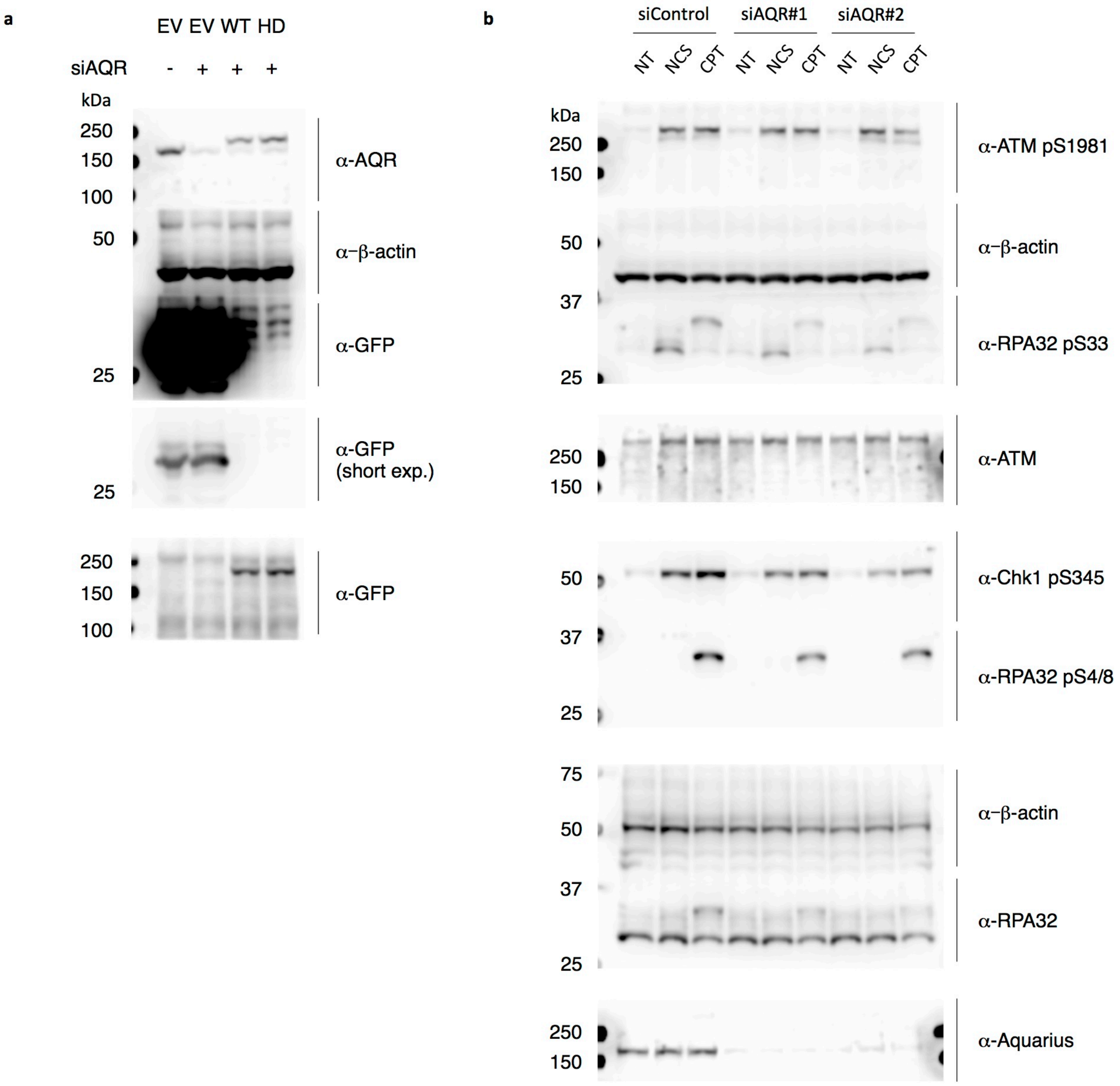

Figure S4
Sakasai et al. Aquarius is required for proper CtIP expression and homologous recombination repair.

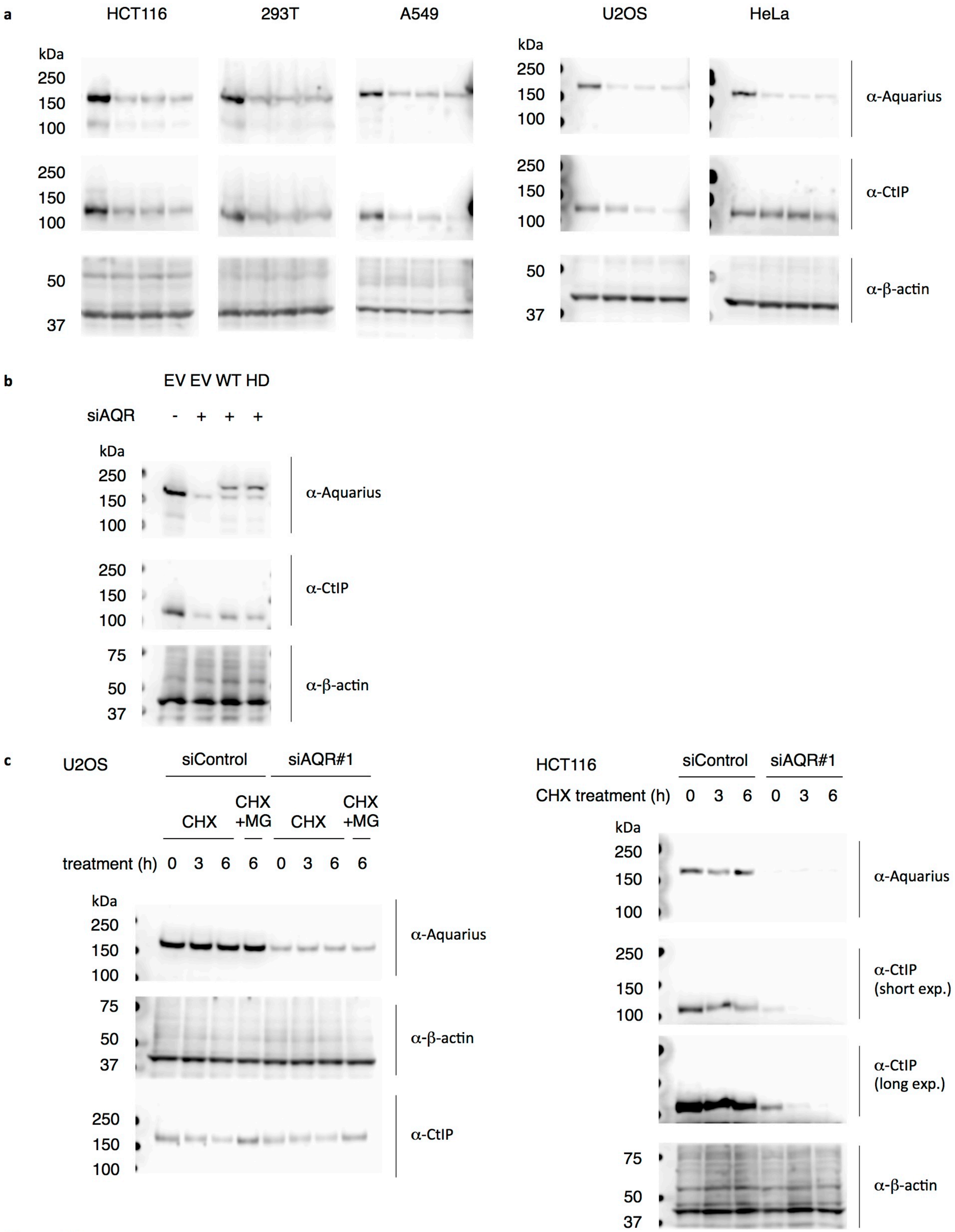

Figure S5
Sakasai et al. Aquarius is required for proper CtIP expression and homologous recombination repair.
